# Metagenome of Gut Microbiota Provides a Novel Insight into the Pathogenicity of *Balantioides coli* in Weaned Piglets

**DOI:** 10.3390/ijms241310791

**Published:** 2023-06-28

**Authors:** Kai He, Jie Xiong, Wentao Yang, Lizhuo Zhao, Tianqi Wang, Weifeng Qian, Suhui Hu, Qiangqiang Wang, Muhammad Tahir Aleem, Wei Miao, Wenchao Yan

**Affiliations:** 1 Parasitology Laboratory, College of Animal Science and Technology, Henan University of Science and Technology, Luoyang 471023, China; 2 National Animal Protozoa Laboratory, College of Veterinary Medicine, China Agricultural University, Beijing 100193, China; 3 Key Laboratory of Aquatic Biodiversity and Conservation, Institute of Hydrobiology, Chinese Academy of Sciences, Wuhan 430072, China; 4 Center for Gene Regulation in Health and Disease, Department of Biological, Geological, and Environmental Sciences, College of Sciences and Health Professions, Cleveland State University, Cleveland, OH 44115, USA

**Keywords:** weaned piglets, *Balantioides coli*, metagenomic analysis, gut microbiota, diarrhea

## Abstract

*Balantioides coli* plays an important role in the diarrhea of weaned piglets, but its pathogenic potential and interaction with gut microbes remain unclear. To investigate the impact of *B. coli* colonization on the gut bacterial structure and function of weaned piglets, a metagenomic analysis based on shotgun sequencing was performed on fresh fecal samples collected from ten *B. coli*-colonized piglets and eight *B. coli*-free ones in this study. The results showed that decreasing diversity and shifted composition and function of the bacterial community were detected in the weaned piglets infected by *B. coli*. In contrast to the *B. coli*-negative group, the relative abundances of some members of the *Firmicutes* phylum including *Clostridium*, *Ruminococcus* species, and *Intestinimonas butyriciproducens*, which produce short-chain fatty acids, were significantly reduced in the *B. coli*-positive group. Notably, some species of the *Prevotella* genus (such as *Prevotella* sp. CAG:604 and *Prevotella stercorea*) were significantly increased in abundance in the *B. coli*-positive piglets. A functional analysis of the gut microbiota demonstrated that the differential gene sets for the metabolism of carbohydrates and amino acids were abundant in both groups, and the more enriched pathways in *B. coli*-infected piglets were associated with the sugar-specific phosphotransferase system (PTS) and the two-component regulatory system, as well as lipopolysaccharide (LPS) biosynthesis. Furthermore, several species of *Prevotella* were significantly positively correlated to the synthesis of lipid A, leading to the exporting of endotoxins and, thereby, inducing inflammation in the intestines of weaned piglets. Taken together, these findings revealed that colonization by *B. coli* was distinctly associated with the dysbiosis of gut bacterial structure and function in weaned piglets. Lower relative abundances of *Clostridiaceae* and *Ruminococcaceae* and higher abundances of *Prevotella* species were biomarkers of *B. coli* infection in weaned piglets.

## 1. Introduction

*Balantioides coli* (formerly Balantidium coli) is a globally distributed waterborne zoonotic protozoan parasite that is mostly in tropical and subtropical regions. With respect to its pathogenicity, it has largely been considered a leading factor in the development of diarrhea for different animals and humans [1]. Animals are initially infected by ingesting contaminated food or water with cysts of *B. coli*, which raise the parasite invasion for the intestine epithelium and contribute to gut dysfunction accompanied by serious diarrhea eventually [2]. Diarrhea has been viewed as one of the most frequently stated problems during the growth and development of pigs [3,4]. Particularly, it poses a serious threat to piglets’ health, with a mortality rate previously reported to be 49% [5], thereby seriously affecting the economic benefits of the pig industry. Therefore, it is essential to advance the understanding of the specific microbial pathogens responsible for triggering piglet diarrhea.

The commensal microbiota are microbial communities that reside in the intestines of animals and play a vital role in the growth, metabolism, and immune status of the host [6,7]. The structure and function of the microbial community can be disrupted by pathogens including bacteria, viruses, and parasites, which usually results in a reduction in probiotics and blooming of harmful bacteria in the gut environment. For example, numerous studies regarding viral-infection-induced dysbiosis in the gut of piglets have been widely reported. Porcine transmissible gastroenteritis virus (TGEV), porcine epidemic diarrhea virus (PEDV), and porcine rotavirus (PoRV) are the most common viral pathogens causing piglet diarrhea [8,9]. A previous study [10] suggested that diarrhea induced by PEDV infection was accompanied by a markedly increased abundance of pathogenic bacteria, including *Escherichia-Shigella* and *Fusobacterium*, in infected piglets. It is a well-known fact that diarrhea is mainly caused by parasitic protozoans in a large number of cases. Parasitic infections have become a potential threat to the health of hosts, but limited information related to their interactions with gut microbes is available. Alternatively, much work has been performed in recent years to elucidate the interactions among parasite infections, intestinal microbiota, and hosts. Evidence is mounting that many enteric parasites could contribute to marked changes by shaping the gut microbial community in hosts [11]. For instance, *Toxoplasma gondii*, a protozoan parasite that mainly leads to foodborne illness, causes intestinal dysbiosis by increased facultative anaerobes, such as some members of the Enterobacteriaceae family, during invasion [12]. Several studies on the enteric protozoa *Blastocystis* have linked significantly increased diversity of human gut microbiota, which is contributed primarily by the members of *Clostridia* class [13]. These findings increasingly suggest that the gut microbiota may have a crucial influence on the disease progression of enteric protozoa pathogens.

The microbiome has received increased attention from researchers in a number of disciplines because of the rapid development of high-throughput sequencing over the past ten years. For example, type-2 diabetes (T2D) [14], obesity [15], and some related metabolic diseases [16,17], which are epidemical worldwide, have been proved to have a strong association with the gut microbiota in a host through increasingly mature sequencing strategies [18]. Metagenomics allow us to comprehensively study all microorganisms in the environment without relying on cultivation [19,20,21]. At present, this technology has been applied to various fields, including deep-sea microbes and animal digestive tracts, and gradually promotes our understanding of the microbial ecosystems of various complex environments [22,23].

Up to now, most of the previous studies on *B. coli* have concentrated on morphological observation and potential drug treatment, as well as sequence-based phylogeny analyses, without exploring the relationship between the gut microbiota and *B. coli* colonization [1,2,24]. Although our preliminary cross-sectional study suggested that the alteration in the fecal microbial community in weaned piglets was associated with natural infection with *B. coli* via 16S rRNA next-generation sequencing (NGS) [25], knowledge of the individual microbial species involved in key functions remains limited. To the best of our knowledge, the amplicon-sequencing technique is widely used due to its high efficiency and low cost; however, its inevitable defect is the bias introduced during 16S rRNA amplification [26]. Therefore, a shotgun metagenomic analysis of the microbial community of fecal samples allows us to deeply explore the microbiota inhabiting fecal samples, as well as to reveal the associated genes from a functional point of view. In addition, it is helpful to promote further understanding of *B. coli* and gut–bacterial interactions and to provide novel insight into the pathogenicity of *B. coli* in weaned piglets.

## 2. Results

### 2.1. Enteric Pathogen Identification

Moving trophozoites of *B. coli* were observed in 21 of 30 fresh fecal materials. Of the 21 *B. coli*-positive samples, 20 *B. coli*-positive samples were diarrheal feces, and only one *B. coli*-positive sample was a normal fecal sample without diarrhea. No other parasite was observed in these samples. RT-PCR was utilized to identify whether three common enteric viruses (PEDV, PoRV, and TGEV) were likely to be correlated with diarrhea. The results showed that the PoRV and TGEV viruses were negative in all the samples, while PEDV was positive in one sample of group D and one sample of group H (Supplementary Table S1), which further suggested that *B. coli* may play an essential role in the etiology of weaned piglets’ diarrhea in the study.

### 2.2. Summary of Sequencing Data for the Fecal Microbiota

A total of 120.94 Gb high-quality data were obtained by filtering low-quality reads, as well as adapter and pig DNA contamination, from the Illumina raw data, which accounted for 6–10.5% of the sequencing reads. The actual insert size of our PE library ranged from 318 bp to 381 bp. For each metagenomic sample, on average, 6.72 ± 0.34 Gb clean reads were generated ultimately. After de novo assembly and the gene prediction process, the final constructed nonredundant gene catalog for all 18 samples contained 3,207,206 microbial genes with an average length of 612 bp. Additionally, the statistical information for the assembled contigs and predicted ORFs of each sample is also exhibited in Supplementary Table S2.

### 2.3. Diversity and Composition of Fecal Microbiota between B. coli-Positive and B. coli-Negative Piglets

**Figure 1:**
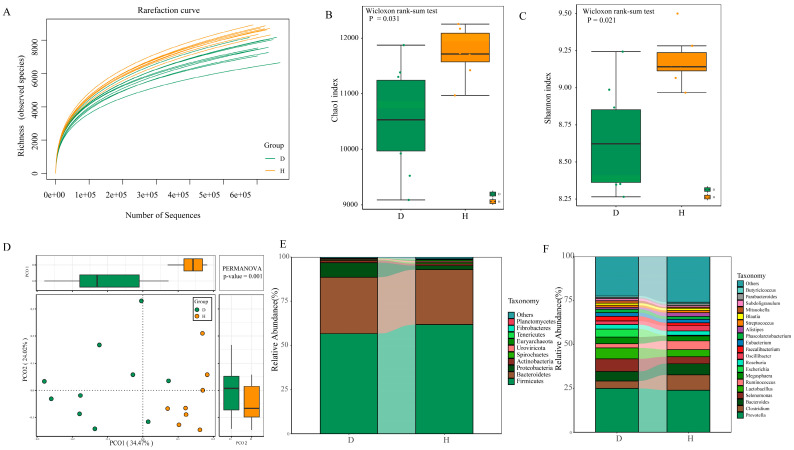
Rarefaction curve (**A**) of the observed species for the 18 samples according to sequencing depth. Significant differences (*p* < 0.05; Wilcoxon rank-sum test) of Chao1 (**B**) and Shannon diversity indices (**C**) are represented using boxplots of the D and H groups. PCoA (principal coordinates analysis) at the species level using compositional profiles based on the Bary–Curtis dissimilarity index was visualized (Adonis *p* = 0.001). The boxes on the top and right represent the first dimension and second dimension between the D and H groups, respectively (**D**). The mean relative abundance of the top 10 phyla (**E**) and top 20 genera (**F**) present in the D and H groups. Microorganisms with a relative abundance lower than 0.1% were classified as “Others”. Group D: *B. coli*-positive piglets with diarrhea. Group H: *B. coli*-free individuals without diarrhea.

The alpha diversity rarefaction curve suggested that the sequencing depth covered the full range of richness for each sample in this study (Figure 1A). The Chao1 and Shannon indices both pointed toward significantly lower microbial diversity in *B. coli*-positive subjects (*p* = 0.003 and *p* = 0.001, respectively) (Figure 1B,C). A principal coordinates analysis showed significant differences in microbial composition between the D and H groups (Figure 1D), as determined using PERMANOVA through the vegan library in R software (www.r-project.org), where *R* = 0.235 (*p* = 0.001). A similar component contribution was observed between the two groups, with PC1 accounting for 34.47% and PC2 accounting for 24.02%.

**Figure 2:**
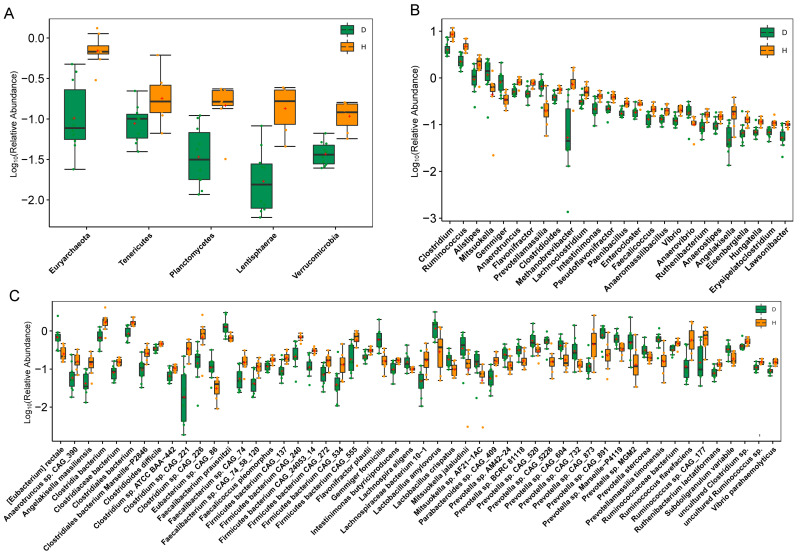
Comparison of gut microbial community of diarrheic weaned piglets infected with *B. coli* and *B. coli*-negative piglets. Boxplots for the differentially enriched phyla (**A**), genera (**B**), and species (**C**) were identified through Wilcoxon rank-sum test (FDR < 0.05, corrected by Benjamini and Hochberg for multiple comparisons). Boxes represent the interquartile ranges, lines inside boxes represent medians, and “+” denotes means. The green and yellow dots represent the biological replicates in Group D and H, repressively. Group D: *B. coli*-positive piglets with diarrhea. Group H: *B. coli*-free individuals without diarrhea.

For the entire microbial composition for each group at the phylum level, *Firmicutes*, *Bacteroides*, and *Proteobacteria* represented major constituents of the gut microbiota and accounted for more than 95% of the total microbes (Figure 1E). However, only five phyla, including *Euryarchaeota* (0.75%), *Tenericutes* (0.22%), and *Lentisphaerae* (0.16%), showed a lower relative abundance in the *B. coli*-positive group (FDR < 0.05) (Figure 2A). At the genus level, a total of 3038 genera (apart from unclassified microorganisms) were identified from all the samples. *Prevotella* was the most abundant genus in the *B. coli*-positive samples (24.0%), followed by *Clostridium* (8.76%) and *Bacteroides* (6.32%) (Figure 1F). We observed that the abundances of 26 genera were significantly changed as a result of *B. coli* infection (Figure 2B). Among them, *Anaerovibrio*, *Gemmiger*, *Mitsuokella*, and *Prevotella massilia* exhibited a higher relative abundance in *B. coli*-infected piglets than in healthy controls (FDR < 0.05). In particular, some bacteria closely related to short-chain fatty acids were observed to decrease significantly in *B. coli*-infected piglets compared to healthy piglets. For example, the relative abundance of *Clostridium* and *Ruminococcus* decreased by 4.54% and 2.56% in group D compared to group H, respectively (FDR < 0.05). In addition, taxonomic community composition profiles at the class, order, and family levels were also identified, and the relative abundances of 3 classes, 5 orders, and 12 families exhibited significantly differences between the D and H groups (Supplementary Table S3). 

At the species level, we firstly used a heatmap plot to focus on the variation in relative abundance for the top 20 species across the 18 fecal samples (Supplementary Figure S1). Among these microbes, *Escherichia coli*, an opportunistic pathogenic bacterium belonging to *Proteobacteria*, exhibited high expression levels in some individuals infected by *B. coli*, which suggests that *E. coli* could be an accidental factor not associated with the development of diarrhea in weaned piglets. However, *Faecalibacterium prausnitzii* within the phylum *Firmicutes*, a well-known butyrate-producing bacterium, exhibited an obviously higher abundance in the D group (FDR < 0.05) (Figure 2C). Using the Wilcoxon rank-sum test, there were 21 increased species and 31 repressed species detected in group D (FDR < 0.05). For the common biomarkers associated with butyrate production, except for some *Clostridium* spp. and *Ruminococcus* spp., *Intestinimonas butyriciproducens* were observed to be enriched in the H group. In addition, the ten species of *Prevotella* from the *Bacteroidetes* phylum were also differential features of the microbiota from piglets with *B. coli*, such as *Prevotella* sp. CAG:604, *Prevotella* sp. MGM2, and *Prevotella stercorea*. Furthermore, the LDA score was calculated at the various taxonomic levels between the two groups through a linear discriminant analysis effect size (LEFSe) analysis, with a threshold above 2.0 identified as being different (see Supplementary Table S3).

### 2.4. Interactions among Enriched Differential Species of Gut Bacteria

**Figure 3:**
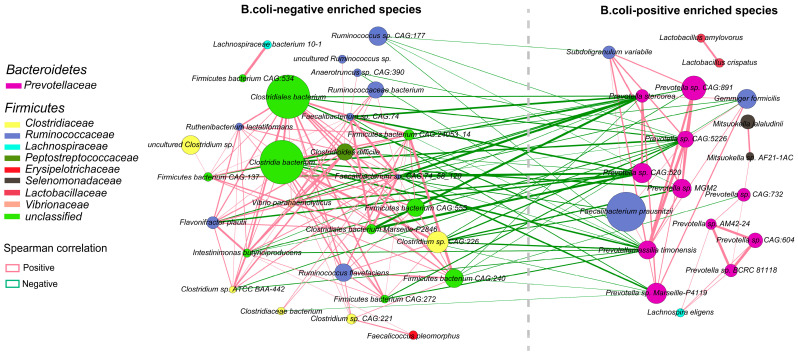
Co-occurrence networks of bacterial species marked differentially enriched in *B. coli*-positive and *B. coli*-negative piglets. The sizes of nodes, which are colored according to family, indicate their mean relative abundance. The thickness of connections between paired nodes is proportional to the Spearman correlation coefficient value (red edges, *R* > 0.8, FDR < 0.05; green edges, *R* < 0.8, FDR < 0.05).

Co-occurrence networks targeting the above-identified 52 markedly altered species between the two groups were constructed to depict the possible relationships of intricate symbiosis and mutual exclusion (Figure 3). Two distinct types of cluster patterns were apparent from the network, which comprised 48 nodes and 178 edges. 

On the whole, the results showed that the majority of the species belonging to *Bacteroidetes* phyla were significantly enriched in *B. coli*-infected piglets compared to healthy piglets. On the contrary, the species decreased in group D were from various butyrate-producing bacteria, including numerous members of *Clostridiaceae* and *Ruminococcaceae*. It was clearly observed that there was a negative association between the two groups and a positive association within the groups, shown by the green and red lines, respectively. The most obvious example was the negative correlation between several *Prevotella* spp. and *Clostridium* sp. CAG:226 (*R* < −0.8, *p* < 0.05) between the D and H groups. Interestingly, there was a strong symbiotic relevance between the two species of *Lactobacilus* (*R* = 0.936), as well as two species of *Mitsuokella* (members of *Selenomonadaceae*) (*R* = 0.902), but nearly no correlation with other species in the D group. There were more positive correlations among microbes shown in the H group. For example, two species of *Clostridia* located at the center of the network were positively correlated with at least 10 other members belonging to *Firmicutes*. Furthermore, detailed information about the correlations among the differential microbes in the network is listed in Supplementary Table S4.

### 2.5. Comparison of Functions of Gut Microbiota in B. coli-Colonized and B. coli-Free Piglets

**Figure 4:**
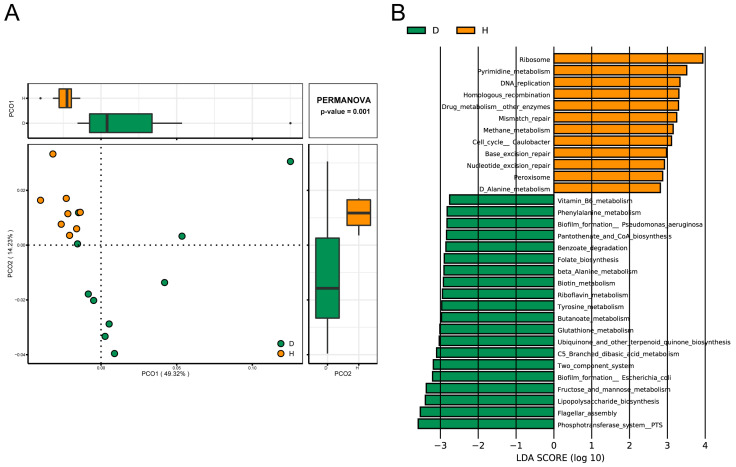
(**A**) The principal coordinates analysis (PCoA) of the metagenomic samples based on the KO profiles. (**B**) The significantly different rankings associated with KEGG pathways involved in Metabolism, Cellular processes, and Environment and Genetic Information Processing were performed using a LEFSe analysis.

By querying the acquired nonredundant protein sequences against the KEGG and EggNOG databases using Diamond software (www.crystalimpact.com/diamond), 5583 KEGG orthologues (KOs) and 3818 EggNOG orthologue groups (OGs), which covered 33.9% and 70.5% of the genes in the gene catalog, were identified, respectively. To elucidate the distribution of samples based on the normalized read abundances of KOs, samples were clustered distinctly into two populations between the two groups by a PCoA, and 49.32% variation was explained in the first principal component (Figure 4A). At a significant cutoff of FDR < 0.05, a total of 275 KOs and 217 OGs showed strikingly different enrichment in the two groups. The results showed that some KOs or OGs linked to the process of transport and oxidation reduction were elevated in the D group. In particular, we noticed that the abundances of several OGs related to the synthesis of lipid A (COG1560), the phosphotransferase system (PTS) (COG1299, COG3731, and COG3444), and some secretion system proteins (COG4796 and COG3504) were higher in the D group, which likely affected the proliferation and endotoxin secretion of bacteria. 

More precisely, the differential third-level pathways belonging to four first-level components of “Metabolism”, “Cellular processes”, “Genetic information processing”, and “Environmental information processing” were further identified via a linear discriminant analysis (LDA) plot (Figure 4B). A significant shift in the abundance of 32 metabolic pathways was detected to be associated with *B. coli* infection, where 20 pathways showed higher expressions in group D (Supplementary Table S5). For example, compared with group H, a significant reduction in the abundance of ribosome biogenesis and a higher abundance in the PTS, biofilm formation, the two-component system, lipopolysaccharide (LPS) biosynthesis, and flagellar assembly ability was seen in group D. Furthermore, the significantly differential KOs involved in the above-mentioned several key metabolic pathways are listed in Supplementary Table S6. Notably, seven KEGG orthologue markers greatly enriched in group D were related to LPS biosynthesis, which may participate in the formation of lipid A and the outer membrane of bacteria.

**Figure 5:**
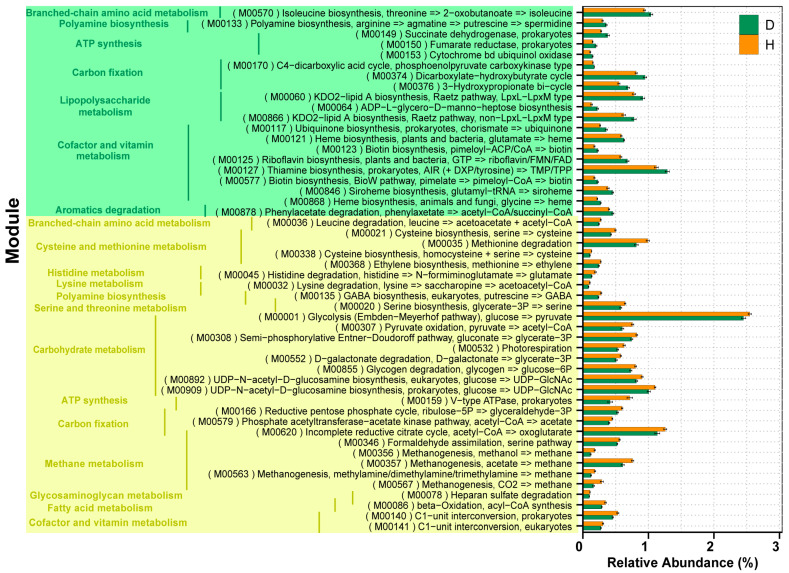
Comparison of gut microbiota KEGG modules between groups D and H. Light green represents the modules with higher abundance in the *B. coli*-positive group; light yellow represents the opposite.

Then, the KOs involved in the functional module levels (small sets of genes in well-defined metabolic pathways) were further investigated, and a total of 302 KEGG modules were identified among all the individuals in the study. There were approximately 50 modules that varied markedly in relative abundance between the two groups (Figure 5, Supplementary Table S7). Compared to the healthy group, there was only one (M00159) of four modules associated with ATP synthesis belonging to energy metabolism reduced in the D group. Another crucial module (M00086) of fatty acid metabolism was also shown to exhibit a lower abundance in the D group relative to the H group, which was capable of mediating the synthesis of acyl-CoA and beta oxidation and forming acetyl-CoA ultimately. For lipopolysaccharide metabolism, relatively high abundances of two modules directly related to KDO2-lipid A biosynthesis (M00060 and M00866) were observed. Furthermore, we found that the module (M00060) was primarily dominated by *Prevotella* and *Bacteroides*, which covered 40.55% and 9.02% of the total genes assigned to the function, respectively (Table 1). Meanwhile, the enriched level of genes in lipid A biosynthesis was positively correlated with the high relative abundance of *Prevotella* and *Selenomonas* (*R* > 0.5, *p* < 0.05) and negatively correlated with *Clostridium* and *Ruminococcus* (*R* < −0.7, *p* < 0.05). 

**Figure 6:**
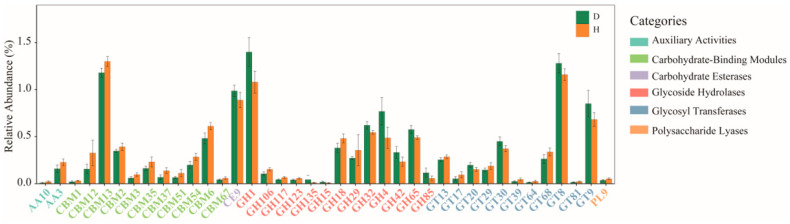
Distribution of categories of differential genes by carbohydrate-active enzymes (CAZymes) database.

In addition, with respect to CAZyme profiles, 8.94% of the predicted gene catalogs were matched to the CAZymes database, in which a total of 209 genes encoding CAZymes were identified (Supplementary Table S8). The obtained CAZymes contained 4 auxiliary activities (AAs), 38 carbohydrate-binding modules (CBMs), 15 carbohydrate esterases (CEs), 44 glycosyl transferases (GTs), 98 glycoside hydrolases (GHs), and 10 polysaccharide lyases (PLs). In group H, genes encoding GT2 (16.51 ± 0.53%) were the most abundant, followed by those encoding GT4 (9.09 ± 0.24%), CBM50 (6.48 ± 0.52%), GH13 (3.29 ± 0.13%), and GH23 (2.78 ± 0.29%). A differential analysis of all the CAZymes was subsequently conducted to investigate the effects of *B. coli* infection on CAZymes. Compared with the CAZymes in the H group, the relative abundances of 39 differential CAZymes were significantly altered in the D group, of which, 13 (1 CE, 8 GHs, and 4 GTs) were upregulated and 26 (11 CBMs, 5 GHs, 7 GTs, 2 AAs, and 1 PL) were downregulated (Figure 6).

### 2.6. Correlation between the Microbial Species and Functions

**Figure 7:**
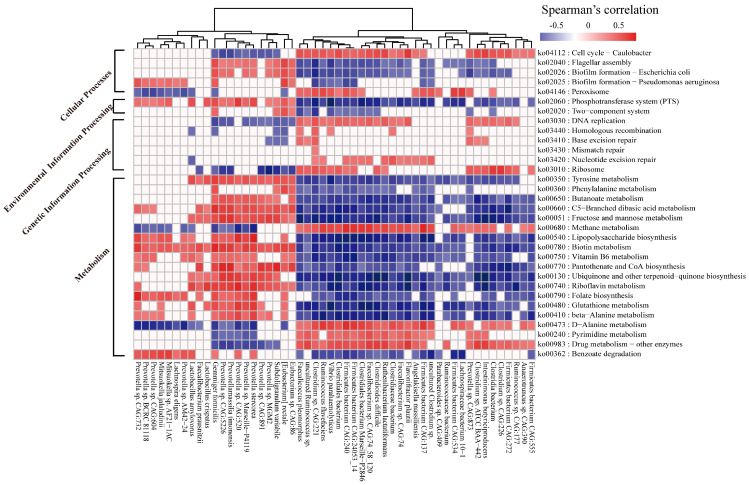
Spearman’s rank correlations were used to show the interactions between biomarkers of KEGG pathway levels and identified key species. Only strong correlations (|*R*| > 0.5, FDR < 0.05) are displayed in the heatmap.

Of particular interest in the association of the above functional features and identified key bacteria, we performed a Spearman’s correlation analysis targeting 32 potential metabolic pathways and 52 differential species (Figure 7). The majority of butyrate-producing beneficial bacteria belonging to *Firmicutes* seemed to have markedly negative correlations with some important pathways, such as the PTS, biotin metabolism, and LPS biosynthesis (*R* < −0.5, *p* < 0.05). Additionally, a significant positive correlation (*R* > 0.7, *p* < 0.05) between *Gemmiger formicilis* and two pathways mainly involved in bacteria motility (ko02040 and ko02026) was observed. Notably, *Gemmiger formicilis* also had a strong symbiotic relationship (*R* > 0.8, *p* < 0.05) with certain species of *Prevotella* (Supplementary Table S4). In addition, Spearman’s rank correlations between the gut microbiota and module-based function were also assessed (Figure S2), aiming to further explore how these bacterial species could affect the downstream function or metabolic substrate. Here, the interactions of microbial species and seven major pathways were concentrated on. Approximately 50 species show significant relationships with two modules (M00064 and M00866) involved in the synthesis of lipid A (*R* > 0.5, *p* < 0.05), with 18 showing positive associations between the bacterial species and M00064. The strongest (*R* > 0.75, *p* < 0.05) were found for four *Prevotella* species, including *Prevotella stercorea*, *Prevotella* sp. CAG:732, *Prevotella* sp. CAG:520, and *Prevotella* sp. Marseille-P4119. On the other hand, a notable feature was also displayed, where the synthesis of acetyl-CoA (M00086) was positively correlated with the majority of members belonging to *Clostridiaceae* and *Ruminococcaceae*, as well as with a member of *Intestinimonas*, which suggests that these microbes were more capable of accumulating short-chain fatty acid (SCFA) concentrations.

## 3. Discussion

The gut is densely inhabited by microbial communities in hosts [27]. In recent years, accumulated evidence has made significant advances in our understanding of how the gut microbiota of animals contribute to health and diseases [28,29]. At the same time, the major members, including bacteria, viruses, and parasitic protozoa, in the intestinal environment have been shown to be capable of invading or modulating the host immune system [30]. However, information on the interactions of protozoan parasites and gut microbiota in hosts remains limited. Therefore, investigating whether or not *B. coli* colonization is correlated with intestinal flora dysbiosis and functional disorder may be important for understanding the pathogenicity of *B. coli*. For this purpose, this study was designed to comprehensively evaluate the effects of *B. coli* on the piglet microbiome by utilizing metagenomic sequencing, which mainly focused on the bacterial species levels and functions. The data presented here illustrate that *B. coli* infection was associated with a significant change in the relative abundance of microbial species and metabolic functions in weaned piglets. These findings may better facilitate our understanding of the interaction between the gut microbial community and *B. coli* colonization in weaned piglets.

Similar to some previous reports on host–parasite systems (except for *Blastocystis*) using the NGS method [11,31], significantly reduced diversity and richness were observed in the *B. coli*-infected piglets by calculating the Chao1 and Shannon indices based on species level in this study. Compared with the *B. coli*-negative piglets, a lower relative abundance of Firmicutes was observed in *B. coli*-positive ones, which is consistent with our previous study [25]. In some human omics studies [14,32], it also has been established that *Firmicutes*, *Bacteroides*, *Proteobacteria*, and *Actinobacteria* are the four most abundant phyla, making up a substantial amount of 98% of the total bacterial community in the gut. Additionally, five nondominant phyla (such as *Planctomycetes*, *Tenericutes*, and *Verrucomicrobia*) exhibited significant differences between the two groups (FDR < 0.05 and LDA > 2) in this study and may act a vital role in the gut microbial community. For example, it was recently shown that several members of *Verrucomicrobia* could be capable of generating glycoside hydrolysis (e.g., lysozyme) to contribute to antibacterial defense strategies [33]. 

Among the genera enriched in both groups, *Prevotella* is widely believed to have a lower abundance in nursing pigs and a higher abundance in weaned piglets [34]. In addition, Karasova et al. [35] observed a reduced relative abundance in diarrheal weaned piglets, which is not consistent with our results in this study. For this phenomenon, a previous study revealed that the microbiota composition could be shaped due to different feeding diets or supplementation during the nursing and weaning periods of piglets [36]. In addition, the presence of *B. coli* could also be a potential factor to impact the abundance of some crucial bacteria in our study. In fact, emerging reports have suggested that at least certain species of *Prevotella* exhibit clinically significant pathogenic properties due to the high genetic diversity within and between species [37,38], which is possibly related to inflammation disorders. Therefore, in-depth metagenomic characterization of microbiota at the species level is needed to explain the disease-regulating properties of *Prevotella*. 

In the present study, a strong negative correlation between ten species of *Prevotella* and a majority of the species belonging to *Clositria* was displayed through the constructed network, which is in agreement with a study where *Prevotella*-dominated bacteria generally was reduced in a beneficial microbial community primarily composed of *Clostridia* cluster XIV and *Lachnospiraceae* clades under inflammatory conditions [39]. Moreover, several reports have suggested that *Clostridium* spp. of clusters XIV and IV are conducive to inducing the accumulation of colonic T regulatory cells (Treg), which play some crucial roles in the balance of gut and immune homeostasis, contributing to the control of some intestinal inflammation and maintaining tolerance toward the gut microbiome [40,41]. Further investigation into the roles of these bacteria in disease and health in hosts is needed.

A similar essential function could be mediated by *Intestinimonas butyriciproducens* due to the ability to produce butyric acid, as reported in previous studies [42]. Intriguingly, *Faecalibacterium prausnitzii* enriched in the *B. coli*-positive group is also a well-known butyrate-producing bacteria that is commonly considered as a clinical biomarker, accompanied by a reduced abundance in patients with most intestinal inflammation diseases, such as Crohn’s disease [43]. The reason could be explained by the significantly negative association (*R* < −0.8, *p* < 0.05) between *Clostridium* sp. CAG:226 (one member of *Clostridia*) and *F. prausnitzii* observed in our current study (Supplementary Table S4), although the association has not been reported in previous studies. Altogether, the data demonstrated that the diarrheic piglets with *B. coli* had a distinct gut microbiome signature compared to normal piglets without diarrhea, which supports our opinion that *B. coli* could be considered a contributing factor in the diarrhea process. 

As the outcome of metabolic function differences between the two groups, increased relative gene abundance for lipopolysaccharide (LPS) metabolism was observed in the *B. coli*-positive group. A recent study [28] revealed the LPS was a major component of the outer membrane of some opportunistic pathogens called Gram-negative bacteria, contributing to the onset and progression of inflammatory response to aggravate the related diseases. Notably, lipid A is the innermost of the three regions of an LPS, held responsible for the endotoxin activity of bacteria [44]. At the module level, two modules involved in lipid A synthesis (M00060 and M00866) were both enriched in the *B. coli*-positive group. Furthermore, 10 identified *Prevotella* species had a strongly positive correlation with the module function, which may provide evidence for the previous conclusion that most of the members belonging to Bacteroidetes are LPS producers in the gut [45]. Therefore, these findings further suggested that the alteration in species of *Prevotella* in the study may confer a higher risk for causing intestinal damage and triggering diarrhea.

In addition, two important pathways were significantly enriched in the *B. coli*-infected group: biofilm formation (1.24-fold) and flagella assembly (1.47-fold). Previous studies revealed that the development of a biofilm may allow for an aggregate cell colony (or colonies) to be increasingly tolerant or resistant to antibiotics [46]. Relatedly, it is believed that the formation of biofilms could affect many aspects of bacterial lives that are closely related to bacteria chemotaxis and flagella assembly, resulting in a rise in bacteria motility [47]. Alternatively, by utilizing the chemotaxis functions, some flagellated bacteria, such as *Escherichia coli*, sense chemical gradients and move toward environmental conditions they deem favorable and/or far away from surroundings they find repellent [48], playing a vital role in the survival and competition of bacteria. Additionally, several *Prevotella* species were also strongly correlated to the two key metabolic functions (R > 0.5, *p* < 0.05), which further suggested that the piglets infected by *B. coli* may have a better growth environment for some opportunistic pathogenic bacteria. In addition, the pathways of the two-component system and the PTS were also increased in the *B. coli*-infected group, and these pathways are responsible for assisting with signal transduction among bacteria and utilizing multiple energy sources [49,50], which can contribute to the modulation of gene expression.

Three repressed KEGG modules (M00001, M00307, and M00308) related to central carbohydrate metabolism were associated with *B. coli* infection, which were capable of promoting the syntheses of various curial metabolic products including pyruvate, acetyl-CoA, and gluconate. Moreover, a crucial module (M00086) related to fatty acid metabolism also had a greater abundance in healthy piglets, which was involved in the process of converting acyl-CoA to acetyl-CoA through beta oxidation. It is well known that acetyl-CoA enables acetyl to entangle the citric acid cycle (Krebs cycle) to be oxidized for energy production, which suggests that the reduced energy substrate in the weaned diarrheic piglets with *B. coli* is in agreement with published studies [5]. Future studies to measure these key metabolic compounds through metabolomics are needed to validate our data. Furthermore, the annotation results of the CAZymes database provided another piece of evidence that more genes encoding CAZymes (GHs, GEs, PLs, AAs, and CBMs) were enriched in the healthy piglets. In particular, the 11 CBMs belonging to the noncatalytic CAZymes, which hold more capability of degrading complex carbohydrates, were observed to show higher relative abundances than in *B. coli*-positive piglets.

Taken together, the study found a decrease in gut microbial diversity in weaned piglets with *B. coli* compared to healthy piglets, including a decrease in the abundance of *Firmicutes* members with a main depletion of *Clostridium* species and an increase in the abundance of *Prevotella* species, as well as some significantly changed functional signatures. Additionally, subsequent experimental validation using an animal model via fecal microbiota transplantation (FMT) is still needed. Recently, FMT techniques have gained more and more attention from researchers and serve as a novel treatment to perform various inflammatory studies, such as on inflammatory bowel disease (IBD), Behcet’s disease [23], and other metabolic syndromes [51]. In summary, all of these above-mentioned findings not only better facilitate our understanding of the potential colonization consequences of *B. coli* in weaned piglets, but also provide insights into strategies for further exploring the interactions between the gut microbiota and parasites.

## 4. Materials and Methods

### 4.1. Animals and Fecal Sample Collection

The present study followed the Guide for the Care and Use of Laboratory Animals established by the State Council of the People’s Republic of China (issue number 676, revised in 2017) and was conducted in a completely noninvasive manner for the animals. A total of 20 diarrheic and 10 normal fresh fecal samples were collected by rectal swabbing from Duroc × Landrace × Yorkshire crossbred weaned piglets (40–45 days) from a commercial pig farm located in Songxian county of Luoyang in the Henna province of Central China. The symptoms of diarrhea and normal samples were defined based on the criteria in previous research [4]. These piglets were weaned at the same time and fed with the same food and water under a consistent living environment. Collection protocols and processes were carried out in accordance with a previous study [25]. After transporting the fecal samples to a laboratory with dry ice, each material was divided into two portions: one subsample was used for microscopic observation at room temperature, and the other subsample was stored at −80 °C immediately until further processing.

### 4.2. Etiological Identification of Pathogens Causing Diarrhea in Piglets

All fecal samples verified the pathogens leading to diarrhea from two facets, including protozoan parasite and viral infection. On the one hand, the presence of trophozoites or cysts of *B. coli* or other large, enteric parasites was examined through direct smear microscopic observation. For the *B. coli*-positive samples, the number of *B. coli* trophozoites per gram of feces (TPG) was calculated through a limited dilution of 0.5–1 g of feces, as described previously [25]. According to the observations, the diarrheic fecal samples with *B. coli* trophozoites were defined as the D group, while normal fecal samples without *B. coli* were regarded as the H group. Finally, ten samples in group D (TPG ≥ 9000) and eight samples in group H were further screened for the metagenomic analysis (Supplementary Table S1). 

Meanwhile, to verify whether the diarrhea of weaned piglets with *B. coli* was associated with viral infection, RT-PCR assays were conducted for detecting TGEV, PEDV, and PoRV. Total RNA extractions were performed using a TIANamp Virus RNA Mini kit (Tiangen, Beijing, China), followed by cDNA synthesis procedures using a TransScript One-Step gDNA Removal and cDNA Synthesis SuperMix kit (Transgen, Beijing, China) according to the manufacturer’s instructions. In the study, the primer pairs designed by the N genes of PEDV and TGEV and the VP7 gene of PoRV from a previous study [9] were utilized.

### 4.3. DNA Extraction and Metagenomics Sequencing

DNA was extracted from 18 fecal samples using an E.Z.N.A.® DNA Kit (Omega Bio-tek, Norcross, GA, USA) according to the manufacturer’s protocols. The resulting concentration and purity of each sample of DNA were quantified via TBS-380 and a NanoDrop2000, respectively. DNA quality was examined with a 1% agarose gel electrophoresis system. Afterward, DNA was fragmented to an average size of about 300 bp using Covaris M220 (Gene Company Limited, China) for paired-end library construction. Based on the protocols of a TruSeqTM DNA Sample Prep Kit (Illumina, San Diego, CA, USA), some essential steps are indispensable for sequencing, such as adapter ligation. Paired-end sequencing was performed on the Illumina HiSeq 4000 platform (Illumina Inc., San Diego, CA, USA) at Majorbio Bio-Pharm Technology Co., Ltd. (Shanghai, China) using a HiSeq 3000/4000 PE Cluster Kit and HiSeq 3000/4000 SBS Kits according to the instructions (www.illumina.com). All the raw metagenomic sequencing data were deposited into the Genome Sequence Archive of the National Genomics Data Center, China National Center for Bioinformation/Beijing Institute of Genomics, Chinese Academy of Sciences (CRA006926).

To improve the data quality for downstream analysis, SeqPrep (https://github.com/jstjohn/SeqPrep, accessed on 12 October 2022) was firstly used for stripping 3′ adaptors for each data set, followed by filtering with Sickle (version 1.33) to remove the low-quality reads (i.e., length of <50bp, quality value of <20, or having >10 N bases). The remaining reads were aligned with the swine representative genome (assembly Sscrofa11.1) using the Burrows–Wheeler Aligner (BWA) tool (http://bio-bwa.sourceforge.net version 0.7.15, accessed on 15 October 2022), in which any hits associated with the reads and their mated reads were removed.

Filtered clean reads were assembled into contigs for each sample using De-Bruijn-graph-based assembler Megahit software (version 1.1.1, parameters: --min-contig-len 300, accessed on 18 October 2022). The N50 or N90 value was calculated to evaluate the assembly quality. Meanwhile, all the sequencing reads of each sample were mapped to contigs using BWA software (https://bio-bwa.sourceforge.net) to confirm the assembled metagenomic data by calculating the ratio of reads successfully aligned with the contigs divided by the total filtered clean reads, which was defined as Pread_contig (see Supplementary Table S2). Subsequently, assembled contigs were employed for ORF prediction using the MetaGeneMark program (version 2.10, accessed on 22 October 2022). The predicted ORFs with a length of at least 100 bp were retrieved and translated to amino acid sequences via Transeq software (version EMBOSS:6.5.7.0). All sequences from gene sets were clustered in a nonredundant gene catalog using CD-HIT (http://www.bioinformatics.org/cd-hit/, accessed on 3 November 2022) with the threshold of 95% identity and 90% coverage. The gene abundance in each sample was calculated and renormalized to TPM (transcripts per kilobase of exon model per million mapped reads) based on gene length and the sum of reads counts utilizing Salmon (version 1.2.1, accessed on 5 November 2022).

### 4.4. Taxonomic and Functional Annotation from Gut Metagenomes

For the taxonomic annotation, protein sequences of nonredundant gene catalogs were performed using Diamond software (version 0.9.24.125, accessed on 10 November 2022) against the NCBI nonredundant (NR) database at the optimized e-value threshold of 1 × 10^5^. Taxonomic profiles at different levels were obtained with the best hit on the NCBI microbial taxonomy database utilizing TaxonKit software (version 0.2.4, accessed on 11 November 2022). Microbial taxa with a relative abundance of >0.1% in at least 50% of the samples within each group were used for downstream analysis.

Corresponding orthologous group (OG) annotation for the putative amino acid sequences translated from the nonredundant gene catalog was carried out via DIAMOND search against the EggNOG database (evolutionary genealogy of genes: non-supervised orthologous groups, version 5.0, accessed on 13 November 2022). In addition, KEGG orthologue (KO) group assignment and CAZymes annotation were also conducted using the DIAMOND program against the KEGG database (release 88.0) and the carbohydrate-active enzymes database (version 20220731, accessed on 15 November 2022), respectively. Here, the amino acid sequences were mapped to the proteins in the above three databases (KEGG, EggNOG, and CAZyme) with consistent parameters (e-value cutoff of ≤1 × 10^5^ and high-scoring segment pair (HSP) scoring > 60). The KOs, Ogs, and CAZymes with a relative abundance of >0.01% in at least 50% of the individuals within each group were used for the downstream analysis. For each microbial or functional feature (KOs, OGs, and different taxonomic levels), relative abundance was calculated by accumulating the relative abundance of all the genes belonging to the feature.

### 4.5. Construction of Correlation Network

The relationship of either mutualism or exclusion amongst significantly different species of bacteria between *B. coli*-positive and *B. coli*-negative samples was captured through co-occurrence networks. Abundance-based marker species from the metagenomic data were used to calculate Spearman’s correlation coefficient (*R*) using the psych library in R 4.0.2. Only significant correlations with *p* values adjusted using the false discovery rate (FDR) method below 0.05 and absolute *R* values above 0.8 were presented in the network, which was visualized with Gephi 0.9.2.

### 4.6. Bioinformatics Statistical Analysis

Alpha diversity was evaluated using a rarefaction curve and diversity indicators, including the Chao1 and Shannon indices. A principal coordinates analysis (PCoA) was determined based on the Bary–Curtis distance with the abundance of species and KO profiles using the ape package in R. For evaluating case–control differences in the dissimilarity, we performed a permutational multivariate analysis of variance (PERMANOVA) with 999 permutations. Significantly changed microbial or functional features were examined with a nonparametric Wilcoxon rank-sum test (Wilcox.test in R), with the *p* value obtained. *p* values were then adjusted using the Benjamini and Hochberg (BH) method for multiple hypothesis tests in differential abundance. Additionally, a linear discriminant analysis effect size (LEFSe) test that took into account both statistical significance and biological relevance was also performed to identify differential biomarkers of microbial and function. In the study, significant differences were considered as an LDA score > 2 and an FDR value < 0.05.

## Figures and Tables

**Table 1:** Distribution of genes from fecal samples annotated to lipid A biosynthesis and Spearman’s correlations between the module (M00060) and genera.

Module	Genus	Spearman’s Correlation	*p* Value	Number of Genes	Coverage of Genes (%)
M00060: lipid A biosynthesis	*Prevotella*	0.579	0.054	850	40.55
	*Bacteroides*	0.007	0.986	189	9.02
	*Selenomonas*	0.602	0.042 *	123	5.87
	*Phascolarctobacterium*	0.467	0.148	98	4.68
	*Parabacteroides*	−0.222	0.571	48	2.29
	*Mitsuokella*	0.583	0.052	45	2.15
	*Alistipes*	−0.593	0.046 *	44	2.10
	*Megasphaera*	0.600	0.043 *	39	1.86
	*Acidaminococcus*	0.439	0.185	35	1.67
	*Clostridium*	−0.781	0.003 **	33	1.57
	*Ruminococcus*	−0.845	0.001 ***	33	1.57
	*Prevotellamassilia*	0.657	0.022 *	30	1.43
	*Alloprevotella*	0.408	0.229	26	4.24
	*Muribaculum*	0.096	0.883	24	1.15
	*Roseburia*	−0.282	0.448	23	1.10
	*Oscillibacter*	−0.719	0.009 **	22	1.05
	*Eubacterium*	0.292	0.430	21	1.00
	*Schwartzia*	0.067	0.885	20	0.95
	*Dialister*	0.474	0.140	19	0.91
	*Others*			374	17.84

A total of 2096 genes were assigned to the module of lipid A biosynthesis; only genes that could be annotated at the genus level were considered. The increase in abundance of the module was positively correlated with *Prevotella massilia* and *Mitsuokella* and negatively correlated with *Clostridium* and *Ruminococcus*. Significance: * *p* < 0.05; ** *p* < 0.01; *** *p* <0.001.

## Data Availability

All the raw metagenomic sequencing data were deposited into the Genome Sequence Archive of the National Genomics Data Center, China National Center for Bioinformation/Beijing Institute of Genomics, Chinese Academy of Sciences (CRA006926).

## Supplementary Materials

The supporting information can be downloaded at https://www.mdpi.com/article/10.3390/ijms241310791/s1.
