# Supplementary figures and images for: Metagenome of Gut Microbiota Provides a Novel Insight into the Pathogenicity of Balantioides coli in Weaned Piglets
Source: Int J Mol Sci. 2023 Jun 28;24(13):10791. doi: 10.3390/ijms241310791 (PMC10342044; doi:10.3390/ijms241310791)

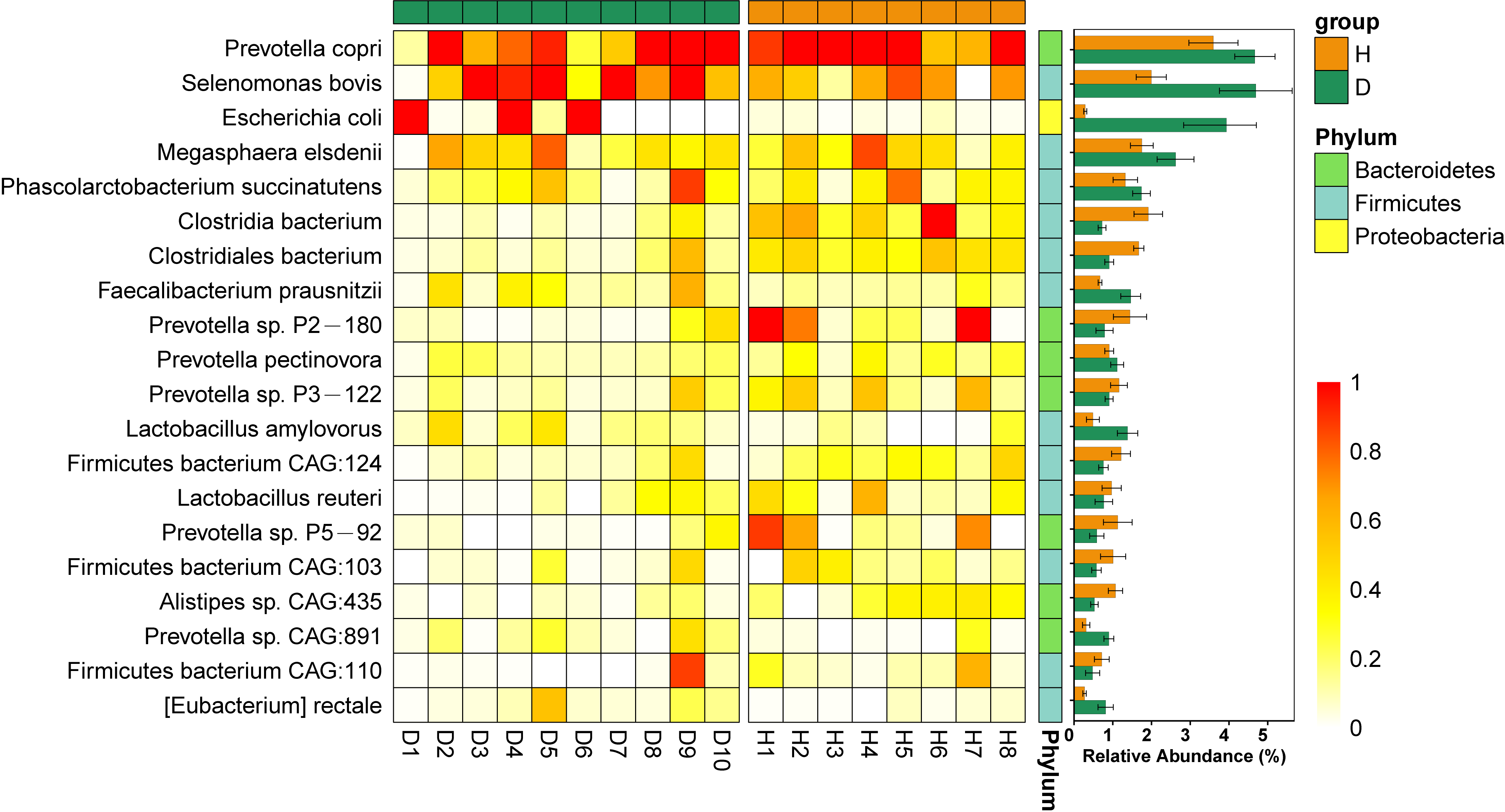

Supplement: Supplementary file 1 [file ijms-24-10791-s001.zip › Supplementary FigureS1.tif]

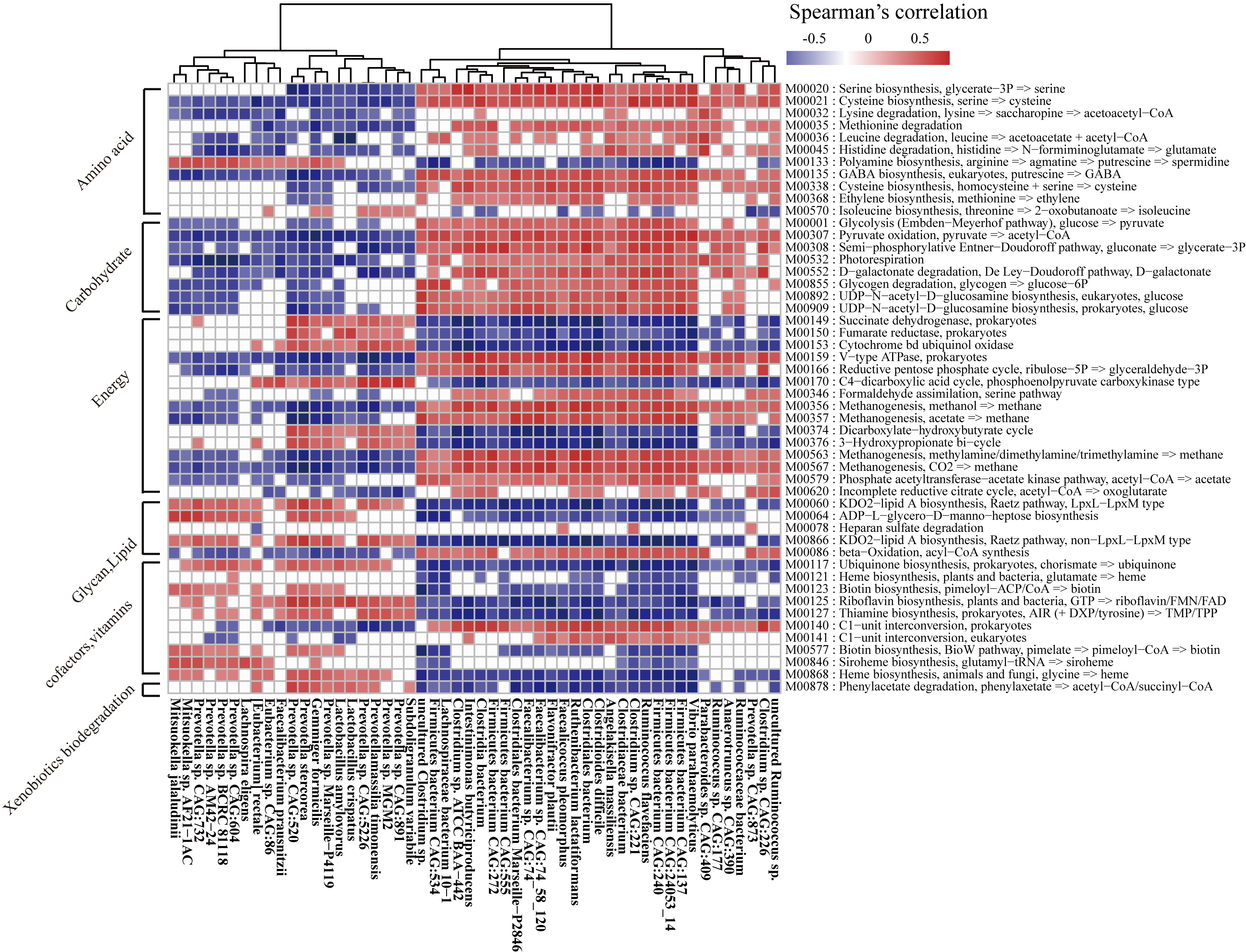

Supplement: Supplementary file 1 [file ijms-24-10791-s001.zip › Supplementary FigureS2.tif]
